# Structure-Based Phylogeny as a Diagnostic for Functional Characterization of Proteins with a Cupin Fold

**DOI:** 10.1371/journal.pone.0005736

**Published:** 2009-05-29

**Authors:** Garima Agarwal, Malligarjunan Rajavel, Balasubramanian Gopal, Narayanaswamy Srinivasan

**Affiliations:** Molecular Biophysics Unit, Indian Institute of Science, Bangalore, India; Institute of Molecular and Cell Biology, Singapore

## Abstract

**Background:**

The members of cupin superfamily exhibit large variations in their sequences, functions, organization of domains, quaternary associations and the nature of bound metal ion, despite having a conserved β-barrel structural scaffold. Here, an attempt has been made to understand structure-function relationships among the members of this diverse superfamily and identify the principles governing functional diversity. The cupin superfamily also contains proteins for which the structures are available through world-wide structural genomics initiatives but characterized as “hypothetical”. We have explored the feasibility of obtaining clues to functions of such proteins by means of comparative analysis with cupins of known structure and function.

**Methodology/Principal Findings:**

A 3-D structure-based phylogenetic approach was undertaken. Interestingly, a dendrogram generated solely on the basis of structural dissimilarity measure at the level of domain folds was found to cluster functionally similar members. This clustering also reflects an independent evolution of the two domains in bicupins. Close examination of structural superposition of members across various functional clusters reveals structural variations in regions that not only form the active site pocket but are also involved in interaction with another domain in the same polypeptide or in the oligomer.

**Conclusions/Significance:**

Structure-based phylogeny of cupins can influence identification of functions of proteins of yet unknown function with cupin fold. This approach can be extended to other proteins with a common fold that show high evolutionary divergence. This approach is expected to have an influence on the function annotation in structural genomics initiatives.

## Introduction

**Figure 1:**
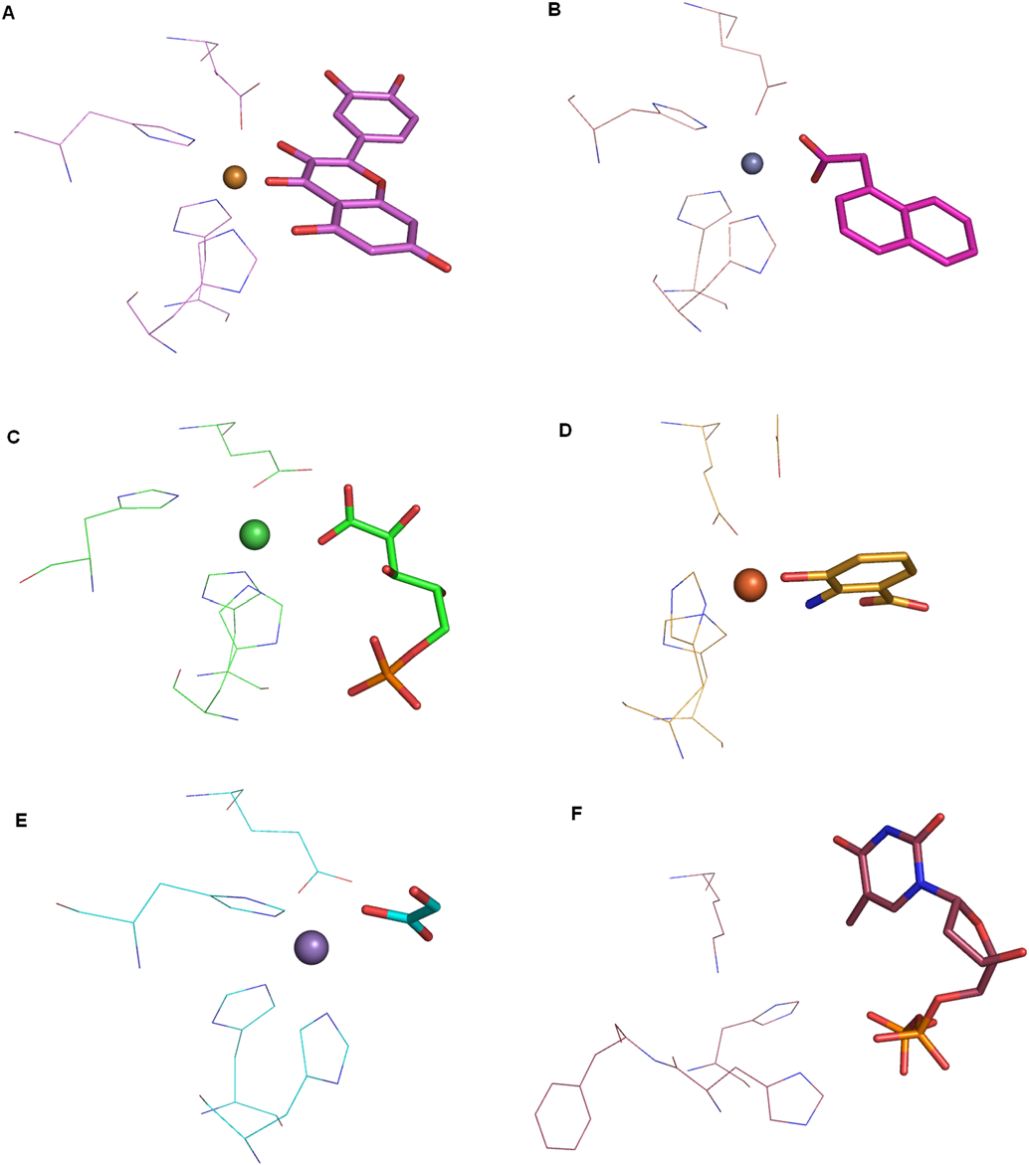
Active site region of various members of cupin superfamily. A. Quercetin dioxygenase (1H1Ia1), B. Auxin binding protein (1LRHa_), C. Glucose-6-phosphate isomerase (1QXRa_), D. 3-hydroxyanthranilate-3,4-dioxygenase (1YFYa_), E. Oxalate Oxidase (2ETEa_) F. RmlC epimerase (1EPZa_). The metals have been shown as spheres, substrates as sticks and metal binding residues as lines. The protein RmlC epimerase do not require metal for its function. All the structural superposition figures have been generated using pymol [16].

‘Cupa’ is a Latin term for small barrel. All proteins that belong to the group of cupins adopt a barrel-like structure [1] . According to the database of Structural Classification Of Proteins (SCOP) [2], the cupin proteins have been classified as members of ‘RmlC-like Cupins’ superfamily in the ‘Double Stranded Beta Helix’ fold. It comprises of 20 families with members performing diverse functions ranging from enzymatic activities like dioxygenases, decarboxylases, hydrolases, isomerases and epimerases to non-enzymatic functions such as binding to auxin, nuclear transcription factors and seed storage. The nature of substrates used in various enzymatic reactions differs in size, chemical types and structural scaffolds. This superfamily is one of the functionally most diverse known thus far [1], [3]. The functional site of members of this superfamily is generally located at the centre of a conserved barrel. Figure 1(A–F) shows the highly conserved location of metals and substrates bound at the active site as well as the spatially conserved metal binding residues in various proteins (See legend for details).

The sequence identity among the members of this superfamily is very low. The typical cupin domain consists of two sequence motifs, each corresponding to two β-strands. These motifs are separated by a less conserved loop region. The conserved motifs, (GX(5)HXHX(3,4)EX(6)G and GX(5)PXGX(2)HXX(3)N, together contain the residues involved in metal ion binding at the active site (typically three histidines and a glutamate), that is known to play a functional role [1], [3]. Full length proteins may contain either one cupin domain (Monocupins) or two cupin domains (Bicupins). In bicupins, the two domains are structurally similar while the sequence similarity can be extremely low. The members of this superfamily also vary in the nature of oligomerization. Extraordinary variations in function, variation in number of cupin domains in the gene products and variation in oligomerization makes this superfamily both challenging and interesting to explore through an evolutionary analysis of structure and function.

As per the statistics derived from the Pfam database [4], 10,346 cupin sequences have been identified in 843 species that belong to eukaryotes, prokaryotes, archaebacteria and viruses. In a few plant species like *Oryza sativa*, *Vitis vinifera* and *Arabidopsis thaliana* more than 100 cupin sequences have been identified. This highlights the extent to which the cupins have duplicated and diverged in proteomes of various organisms to perform diverse functions.

Here we present a 3-D structure-based phylogenetic analysis of cupins and a detailed comparative analysis of structures in order to further our understanding of the relationship between structure and function. We have specifically explored the use of structure-based phylogenetic relationships in deciphering functions of proteins belonging to this superfamily.

## Results and Discussion

### Phylogenetic analysis

It is well known that during evolution, 3-D structure is conserved better than amino acid sequence. Members of cupin superfamily are very diverse and are characterized by low sequence identity among the homologues. Previous studies [5] have shown that in cases of poor sequence identity, structure-based phylogenies generate better models of evolution of proteins than the traditional sequence-based methods. Therefore, this analysis was carried out at the level of 3-D structures of domains (compiled in Table 1). A dendrogram was constructed using a structure dissimilarity matrix obtained from the pairwise structure-based alignment of these proteins at the domain level (See Methods). Since the domains act as functional and evolutionary units, an analysis at the domain level would be more appropriate for studying the structural variations leading to functional variations. Further, studies at the domain level would enhance our understanding of the degree of similarities and differences between the two domains in bicupin members.

**Table 1:** List of proteins used in the present analysis

Sno	SCOP family	Function	Code	Domain nature	Oligomer	Metal*	Reference
1	RmlC Epimerase	dTDP-4-dehydrorhamnose 3,5-epimerase RmlC	1DZT	Monocupin	Dimer	-	[17]
			1EPZ	Monocupin	Dimer	-	[18]
			1NZC	Monocupin	Dimer	-	[19]
			2IXC	Monocupin	Dimer	-	[20]
			2IXH	Monocupin	Dimer	-	[20]
			1WLT	Monocupin	Dimer	-	To be Published
		dTDP-4-keto-6-deoxy-glucose-5-epimerase EvaD	1OI6	Monocupin	Dimer	-	[21]
			2C0Z	Monocupin	Dimer	-	To be published
2	GPI	GPI (Glucose-6-phosphate isomerase)	1QXR	Monocupin	Dimer	Ni	[22]
			1J3R	Monocupin	Dimer	Fe	To be Published
3	Hypothetical protein TM1112	Hypothetical protein TM1112	1O5U	Monocupin	Monomer	-	[23]
4	TM1287-like	Hypothetical protein TTHA0104	1V70	Monocupin	Dimer	-	To be Published
		Hypothetical protein TM1010	2F4P	Monocupin	Dimer	-	To be Published
		Hypothetical protein Spy1581	1YHF	Monocupin	Monomer	-	To be Published
5	TM1459-llike	TM1459-like	1VJ2	Monocupin	Dimer	Mn	[24]
		Hydroxypropylphosphonic acid epoxidase Fom4, C-terminal domain	2BNN	Monocupin	Dimer	Zn	[25]
6	Germin/Seedstorage	Germin oxalate oxidase	2ETE	Monocupin	Hexamer	Mn	[26]
		Auxin binding protein	1LRH	Monocupin	Dimer	Zn	[27]
		Seed storage	2PHL	Bicupin	Trimer	-	[28]
			2CAV	Bicupin	Trimer	-	[29]
			1FXZ	Bicupin	Trimer	-	[30]
			1IPJ	Bicupin	Trimer	-	[31]
			1OD5	Bicupin	Hexamer	-	[32]
			1UIK	Bicupin	Trimer	-	[33]
		Oxalate decarboxylase	1J58	Bicupin	Hexamer	Mn, Mn	[34]
7	Ylba-like	Hypothetical proteinYlba	1RC6	Bicupin	Monomer	-	To be Published
		Glyoxylate-induced protein PA1140	1SQ4	Bicupin	Octamer	-	To be Published
		Hypothetical protein EF2996	1SEF	Bicupin	Monomer	-	To be Published
		Hypothetical protein DR1152	1SFN	Bicupin	Dimer	-	To be Published
8	Pirin-like	Pirin	1J1L	Bicupin	Monomer	Fe,-	[35]
		Hypothetical protein YhhW	1TQ5	Bicupin	Monomer	Cd,-	To be Published
9	Quercetin 2,3-dioxygenase	Quercetin 2,3-dioxygenase	1H1I	Bicupin	Dimer	Cu,-	[10]
			1Y3T	Bicupin	Dimer	Fe,Fe	[6]
10	Phospho mannose isomerase	Phospho Mannose Isomerase	1PMI	Bicupin	Monomer	Zn,-	[36]
			1QWR	Bicupin	Monomer	Zn,-	To be Published
		Putative mannose phosphate isomerase AF0035	1ZX5	Bicupin	Monomer	-	To be Published
11	Homogentisate dioxygenase	Homo gentisate dioxgenase	1EY2	Bicupin	Hexamer	-,Fe	[37]
12	Acireductone-dioxygenase	Acireductone-dioxygenase	1ZRR	Monocupin	Monomer	Ni	[38]
			1VR3	Monocupin	Monomer	Ni	[39]
13	Kdu-like	KduI (5-keto-4-deoxy-uronate isoomerase)	1XRU	Bicupin	Hexamer	-,Zn	[40]
			1YWK	Bicupin	Monomer	-	To be Published
14	Uriedoglycolate hydrolase A11A	Uriedoglycolate hydrolase	1XSQ	Monocupin	Dimer	-	To be published
			2BDR	Monocupin	Dimer	-	To be Published
			1YQC	Monocupin	Dimer	-	[41]
15	Probable transcription factor	Hypothetical protein	1Y9Q	Monocupin	Dimer	Zn	To be Published
16	YML079-like	Hypothetical protein	1XE7	Monocupin	Dimer	-	[42]
		Hypothetical protein Atu3615	1ZNP	Monocupin	Dimer	-	To be Published
		Hypothetical protein SO0799	1YUD	Monocupin	Dimer	-	To be Published
17	PA5104-like	Hypothetical protein PA5104	1YLL	Bicupin	Dimer	-	To be Published
18	MJ0764-like	Hypothetical protein MJ0764	2B8M	Monocupin	Dimer	-	To be Published
19	Cysteine dioxygenase type 1	Cysteine dioxygenase	2ATF	Monocupin	Monomer	Ni	[43]
20	Hydroxy anthranilate dioxygenase	Hydroxy anthranilate dioxygenase	1YFY	Monocupin	Dimer	Fe	[44]

* The presence or absence of metal ion is indicated for both N and C terminal domains in case of bicupins

**Figure 2:**
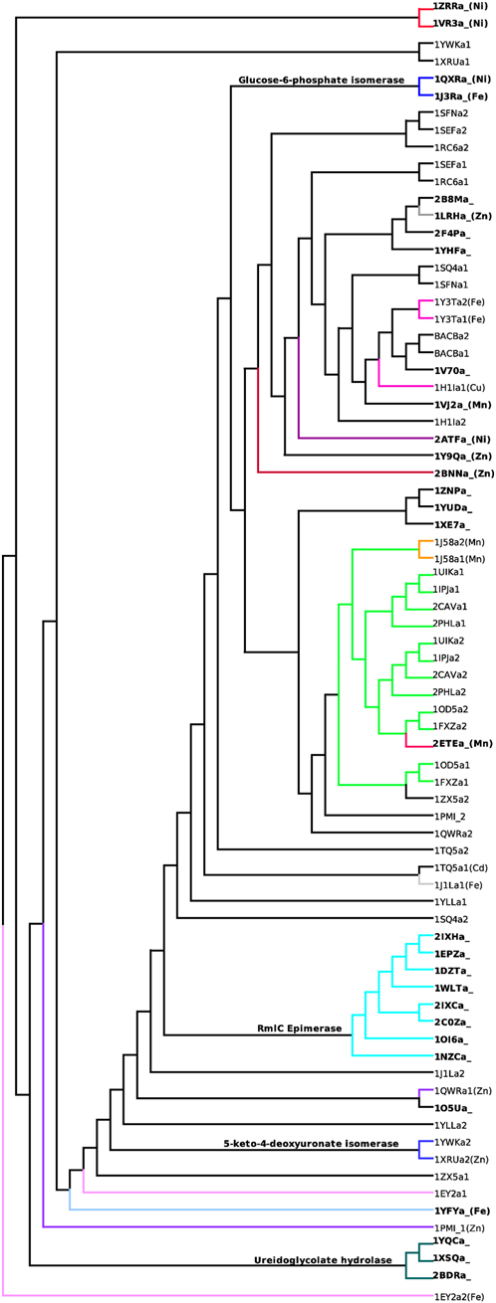
Dendrogram generated on the basis of structural comparisons of cupins at the level of domain folds. The protein domains carrying out similar function are marked in the same colour. The monocupins are shown in bold. The proteins with unknown function are indicated by black lines. The first four letters in the taxon names refer to protein codes, fifth is the chain identifier while the numbers 1 and 2 for bicupins indicate the N and C-terminal domains respectively. The identity of the metal bound at the active site is indicated in brackets. A detailed investigation was performed on the functional clusters indicated in the dendrogram.

Figure 2 shows a dendrogram generated after pairwise comparisons of all the domains considered in this analyses. Although, the dendrogram was generated based on structural differences, functionally similar proteins were observed to cluster. This feature is shown by different colours of the branches in the dendrogram corresponding to different functions. In figure 2, entries of bicupins (two domains of cupin fold within a single polypeptide chain), with information on function available are shown by coloured lines. The hypothetical proteins have been indicated in black lines (Table 1). The taxon labels of monocupins are shown in bold. The metal bound at the active site is mentioned in brackets in the taxon labels. The functional clusters include an eight-membered cluster of the RmlC sugar epimerase family (cyan), three-membered Ureidoglycolate hydrolase (teal), two-membered Acireductone dioxygenase (red), two-membered 5-keto-4-deoxyuronate isomerase (light blue) and two-membered Glucose-6-phosphate isomerase (blue). The dendrogram has two other major clusters where one cluster contains members of germin family (mainly seed storage and oxalate decarboxylase function, marked in green and orange respectively) and C terminal domains of cupins from the phosphomannose isomerase family. The other cluster has mostly hypothetical proteins of yet unknown function and a few characterized members: quercetin dioxygenase (1H1I and 1Y3T, magenta), cysteine dioxygenase (2ATF, dark purple), epoxidase (2BNN, brown) and auxin binding protein (1LRH, dark grey). The N-terminal halves of the proteins which function as phosphomannose isomerase (1PMI_1, 1QWRa1) do not cluster together. A plausible reason for this separation could be the presence of a large α-helical insertion in the N terminal domain of 1PMI (refer to Table 1). The C-terminal domains of the members are similar and co-cluster.

An interesting observation from the structural phylogeny analysis is that although proteins of similar function defined at a finer level (such as binding to common or similar substrates) cluster together, proteins with roughly similar functions (for example, isomerases of different substrates) do not. For example, isomerases like glucose-6-phosphate isomerases (blue), phosphomannose isomerases (purple), 4-keto-5-deoxyuronate isomerase (light blue), rmlC epimerase (cyan) and dioxygenases like cysteine dioxygenase (dark purple), acireductone dioxygenase (red), quercetin dioxygenases (magenta), homogentisate dioxygenase (light pink), hydroxyanthranilate dioxygenase (sky blue) are not localized in the same cluster. In fact, the nodes of these clusters lie far apart in the dendrogram. Thus it is clear that the structure-based phylogeny approach for the cupin superfamily most often tends to cluster proteins of same or similar function if the function is defined at a fine level. To examine this aspect further from the perspective of substrate preferences, we examined the case of quercetin dioxygenase. The enzyme catalyzes the conversion of quercetin to 2-protocatechuolyphlororglucinol carboxylic acid and carbon monoxide (Figure S1). The experimental details on the kinetic data of various substrate preferences for the enzyme in *B. subtilis* are shown in Table S1. The residues involved in positioning the substrate into the active site are highlighted in the structure-based sequence alignment of the known quercetinases (Figure S2). The details of the study and the analysis are discussed in supplementary section (Text S1).

Various metal ions, bound at the active site, including Nickel, Iron, Manganese, Copper, Zinc and Cadmium are known to play a functional role in the enzymatic members of cupin superfamily. The nature of metal ion can influence the chemistry of the catalytic reaction. Therefore, similarities and differences in the property of cupin domains to bind different metals have been discussed for the members of various functional clusters in the dendrogram. The metal ion, wherever known, has been mentioned in brackets in the labels of taxa in the dendrogram (Figure 2). This information is also made available in Table 1.

Overall, the structure-based clustering does not correlate with the identity of the metal bound at the active sites; identical metals are present in many clades that lie far in the dendrogram. However, many functionally similar proteins have identical metals at their active site to bring in the desired reaction mechanism for catalysis. For example, in the domains clustered in the clade corresponding to acireductone dioxygenase (shown in red in figure 2) the metal ion Ni is preserved. Phosphomannose isomerases do not fall in a close cluster but contain Zn at their active sites. Exceptions are the clades of Quercetin dioxygenase and Glucose-6-phosphate isomerases (shown in magenta and blue respectively in figure 2). The enzymes in these two functional clusters have different metals bound at their active sites. Although the metals bound at the sites are different, they can act as electron sinks to allow the catalysis to occur. This has been shown for 1Y3T, a quercetin dioxygenase which elicits enzymatic activity even with different metals at the active site [6]. Crystal structures of metal-bound forms of Ureidoglycolate hydrolases (teal) have not been solved but the metal binding residues are conserved in all the members which indicates the requirement of a metal ion at their active sites. Similar reasoning holds for 1YWK, 5-keto-4-deoxyuronate isomerase for which the structure of the metal-bound form is unavailable. Rmlc sugar epimerase does not require a metal cofactor for activity. It is therefore not surprising that the metal binding residues have not been preserved. The members of Germin family 2ETEa_, an oxalate oxidase and 1J58, an oxalate decarboxylase, have Mn at their active sites. In this context, it is interesting to note that the two proteins act on identical substrates but yield different products.

Generally, the cupin domains of monocupin proteins co-cluster with their monocupin family members (Figure 2, shown in bold). However, in a few cases these domains are interspersed with protein domains that are tethered to another cupin domain. For example, 1VJ2a_ and 2ETEa_ are monocupins but co-cluster with bicupins. This demonstrates high structural similarity between some of the monocupin and bicupin domains and thus their evolutionary relatedness.

In most of the bicupins, N terminal (----a1) and C terminal domains (----a2) share a high similarity to their potential orthologues in other organisms than to each other (paralogues). Examples include members of germin family (green), 4-keto-5-deoxyuronate isomerase-like family (light blue) and hypothetical proteins of ylba-like SCOP family (refer Table 1). Besides, in most of the bicupins, the function has been associated with only one of the two domains while the function of the other domain is unidentified. This has been exemplified through an example of 4-keto-5-deoxyuronate isomerase. The sequence identity between the N terminal domains and between the C terminal domains is about 50%. However, the sequence identity across the two clusters is very low (about 10%). The crystal structures of the two proteins are not available in their substrate bound forms, so the substrate binding residues have not been identified. However, a study of the residues at the active site region located in the C-terminal domain and the degree of conservation around the metal ion shows that metal binding residues (three histidines and a glutamate) and nearby residues tryptophan, methionine, tyrosine, phenylalanine and arginine are conserved while serine is conservatively substituted by threonine (figure not shown). Similarly, the β-barrel fold of N terminal domains of 1XRU and 1YWK are lined by conserved alanine, leucine, lysine, valine, glycine, isoleucine and phenylalanine and are characterized by a few differences such as isoleucine & leucine, glutamate & tyrosine and tyrosine & isoleucine. A comparison of residues across the two clusters reveals the loss of metal binding residues; glutamate and histidine to leucine and the other histidines to valine and glycine. Other residues in the N-terminal domain topologically equivalent to the active site residues of C terminal domain are poorly conserved. A tryptophan has been replaced by tyrosine, methionine by cysteine and tyrosine by valine. A phenylalanine and an arginine are conserved between the N and C-terminal domains of 1XRU. These substitutions suggest the loss of metal binding abilities and degeneration of active site residues in one of the bicupin domain during the evolution of the bicupins. However, the close clustering of these domains implies a conserved, yet unidentified function. These observations indicate an independent evolution of the N and C terminal domains in most of the bicupins. However, the N and C-terminal domains of BacB (uncharacterized) and of 1J58 (oxalate decarboxylase) co-cluster in the dendrogram. The two proteins have been isolated from *Bacillus subtilis* where large scale genome duplication has been reported [7].

In order to gain a better understanding of the structural phylogenetic analysis, these studies were extended to identify structural signals that separate proteins into different clusters in the dendrogram. Since proteins with the same function have clustered, this study could help us to delineate structural signals giving rise to specific functions. The possible structural variations, reflected through SDM measure that could have contributed to branching include insertions and deletions, changes in the lengths or orientations of regular secondary structures and conformational changes in loop regions. This is analogous to the evolutionary trace approach proposed by Cohen and coworkers [8] but performed by considering structural variations between members of the superfamily as opposed to the traditional sequence dissimilarity-based measure. A detailed investigation on the structural differences, of some of the functional clusters indicated in Figure 2 has been carried out.

### Evolutionary trace analysis based on structure-based phylogeny

**Figure 3:**
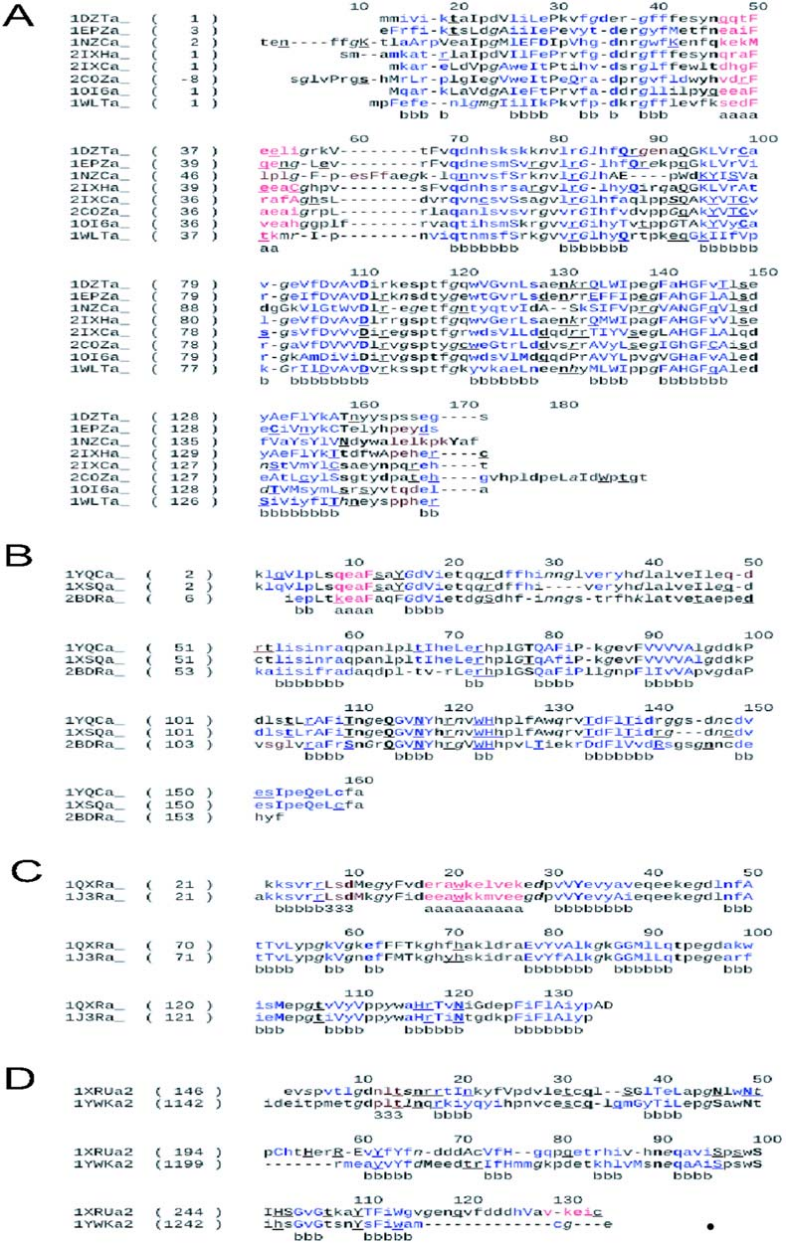
Structure-based sequence alignment of various functional clusters. A. RmlC epimerase (cyan in dendrogram), B. Ureidoglycolate hydrolase (teal in dendrogram), C. Glucose-6-phosphate isomerase (dark blue in dendrogram), D. 4-Keto-5-deoxyuronate isomerase (light blue in dendrogram. The secondary structural elements of the members are highly conserved.

**Figure 4:**
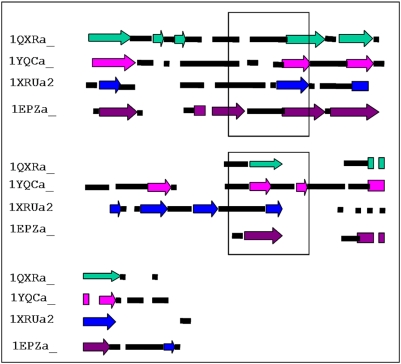
A schematic of the multiple structural alignments of the representative members of each cluster. Helices are shown as rectangles in different colors, strands as arrows and black rectangles are loops. The gaps in the alignment are indicated as white space. The region corresponding to motifs containing metal binding residues has been boxed.

**Figure 5:**
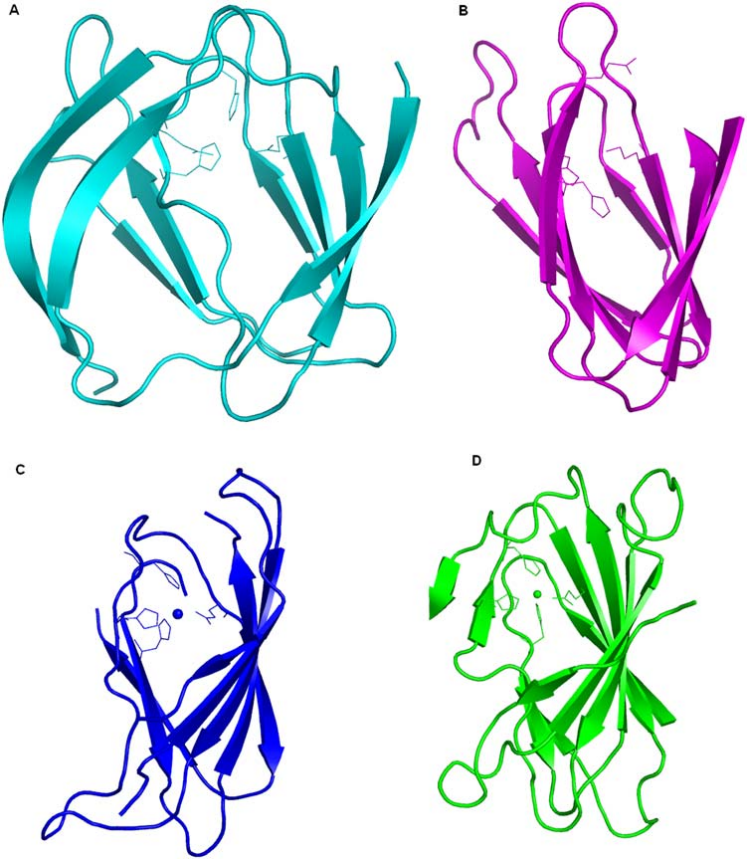
The structural superposition of the core of the proteins. The structure of Ureidoglycolate hydrolase (1YQCa_) is in light blue (A), RmlC epimerase (1EPZa_) in magenta (B), Glucose-6-phosphate isomerase (1QXRa_) in blue (C) and 5-keto-4-deoxyuronate isomerase (1XRUa2) in green (D). The substrates are shown as sticks, metals as spheres and the metal binding residues at the active site as lines. Marked structural variations in the region accommodating metal binding residues can be seen.

Structure-based sequence alignments were generated for members within each cluster. Mapping of secondary structure information onto the alignments using JOY software [9] revealed conservation of secondary structure elements as well as their lengths in the respective families (Figure 3A–D). A multiple alignment of representative members from each cluster and mapping of conserved secondary structure information onto the sequences shows significant variation in the lengths of topologically equivalent β-strands containing the metal binding residues across functionally different cupins (Boxed in Figure 4). The metal cofactor usually plays an important role in the function of cupins through an interaction with the substrate. Substitution of β-strands to loops amongst the members of the superfamily at the active site implies conformational freedom facilitating the binding of diverse substrates (Figure 5A–D). An exception is 1EPZa_ which does not require metal for its function and has lost some of the metal binding residues accommodating more variation in the region.

**Figure 6:**
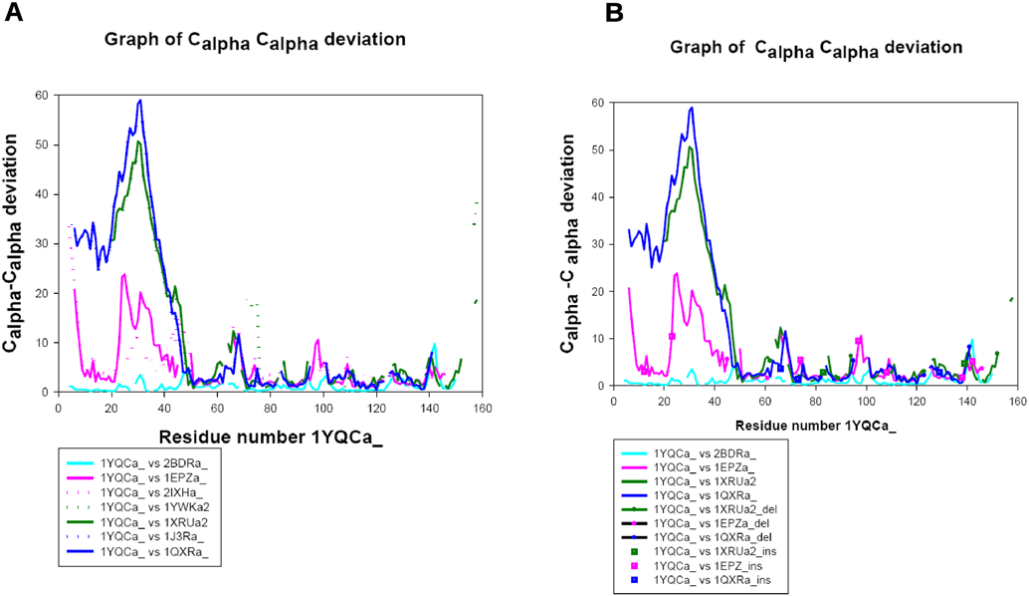
Plots of Cα-Cα deviation versus the residue number of 1YQCa_. A. The Y axis corresponds to the deviation in Cα positions of protein structures when aligned to reference structure, 1YQCa_. The X axis indicates the residue number of 1YQCa_. The proteins performing same function are marked in same color. B. Shows the same plot with deletions and insertions in the proteins with respect to the reference, marked as circles and squares, respectively. The region with very high deviation values has been encircled.

The representative protein structures from the respective clusters were aligned to a reference protein structure of 1YQCa_ and Cα-Cα deviation values were calculated for equivalent residues at all residue-residue alignment positions from the pairwise superpositions. Figure 6A shows a plot of the variation of Cα-Cα deviation values with the residue positions of 1YQCa_. This graph has been color coded to represent different functional classes. Proteins performing the same functions are marked in same colors as solid and broken lines. From the plot it is clear that the proteins belonging to same cluster have similar variations in the Cα-Cα deviation values with respect to the reference protein. The protein 2BDRa_ carries out the same function as 1YQCa_ (cyan) and has the lowest overall deviation values compared to any other protein. RmlC epimerase family and has a lower overall deviation in the plot (1EPZa_ and 2IXHa_, magenta) as compared to other functional clusters. The proteins from the other two clusters (1XRUa2 and 1QXRa_, blue and green respectively) are more similar to each other than the reference. This comes out from the similar values of deviation with respect to the reference in the plot (Figure 2). Figure 6B shows the plot with the representative proteins from each cluster. Any insertions and deletions with respect to 1YQCa_ are marked as squares and circles, respectively (Figure 6B). The lengths of gaps in the line plots indicate the deletion lengths with respect to the reference. Large variation in the values of Cα-Cα deviation for different proteins lie in the regions aligned to the 50 residues of 1YQCa_ (encircled in the plot). These observations suggest that that the structural differences in functional regions discussed above appear to be mainly responsible for the branching in structure-based phylogeny (Figure 2) resulting in different clusters of proteins with different functions.

**Figure 7:**
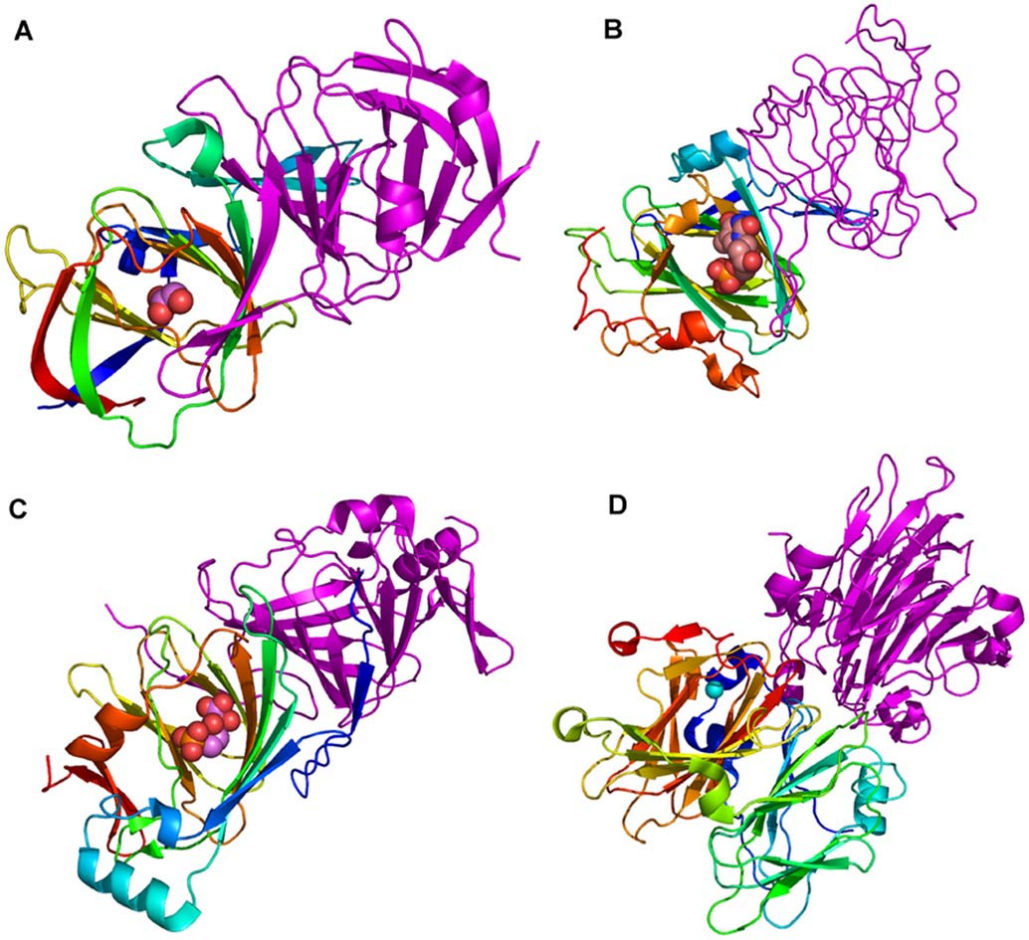
The figures (A–D) show the quaternary structures of the proteins. N to C terminus of one chain of the dimeric proteins has been coloured as spectrum while the other as pink. The ligands at the active site are shown as spheres. A. Ureidoglycolate hydrolase (1YQCa_), the reference protein, bound to the product. The fifty residues in the N terminus are involved in interaction with another subunit in the dimer through domain swapping. The region interacts with substrate in the other subunit. B. RmlC epimerase (1EPZa_) bound to the substrate. The region of large deviation with respect to 1YQCa_ corresponds to the strands forming a beta swapped dimer and covers the active site in the other subunit. C. Glucose-6-phosphate isomerase (1QXRa_) dimer bound to a substrate analog. The structurally variable region lies on the opposite face of the domain in contrast to the reference 1YQCa_ and forms the active site in the same domain. D. 5-keto-4-deoxyuronate isomerase (1XRU) is a dimeric bicupin with Zn bound at the active site. The functional domain considered for comparison lies at the C terminal end. The structurally different region lies at the active site but is not involved in domain swapping with the N terminal domain in the polypeptide.

A closer look at the quaternary structures of these proteins (Figure 7A–D) reveal that these regions are usually interacting with another subunit in monocupins or as a tethered domain in a bicupin (See legend of Figure 7 for details). Also, these regions partially cover the active site pocket either in the domain of the interacting partner or in the same domain.

### Inference on function from structure – implications for structural genomics

**Figure 8:**
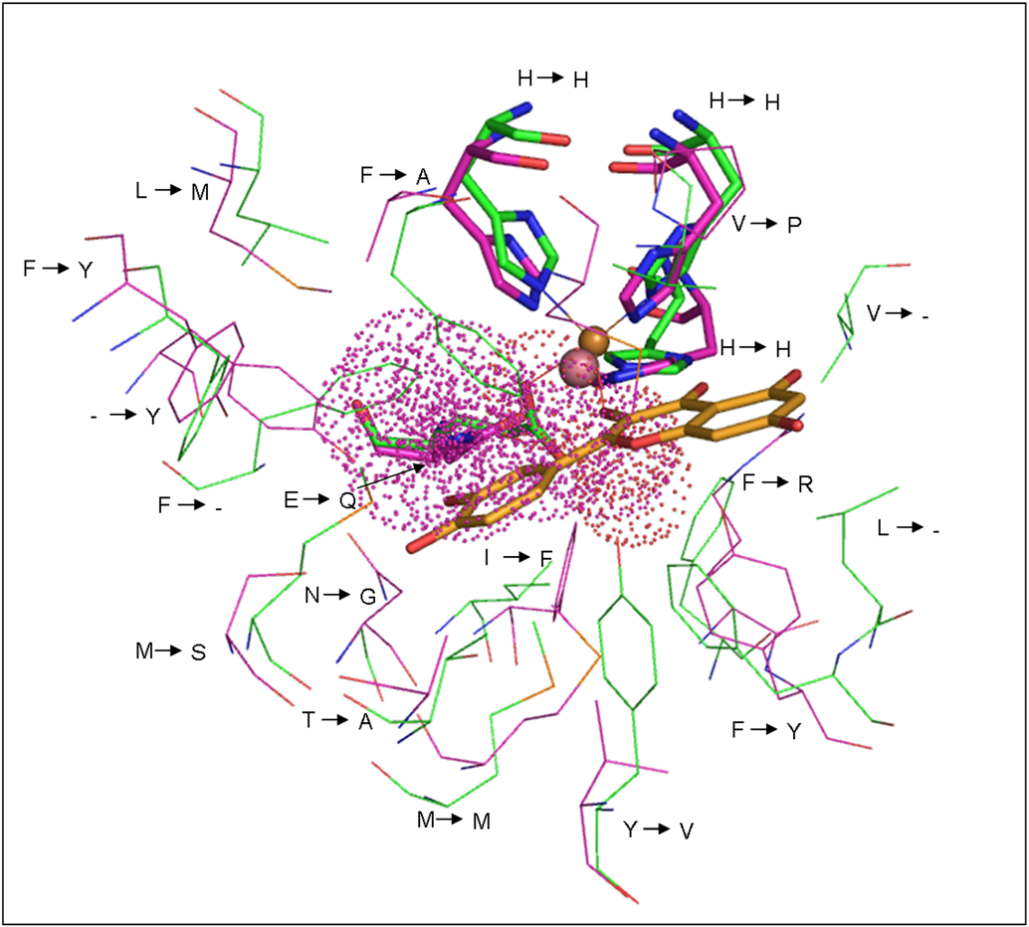
Superposition of the three-dimensional structures of hypothetical protein (BacBa2, magenta) and Quercetinase (1H1Ia1, green). The substitutions at the active site have been indicated. The metal (sphere), metal binding residues (sticks), known substrate (sticks) and the unknown ligand (dots) are also shown.

**Figure 9:**
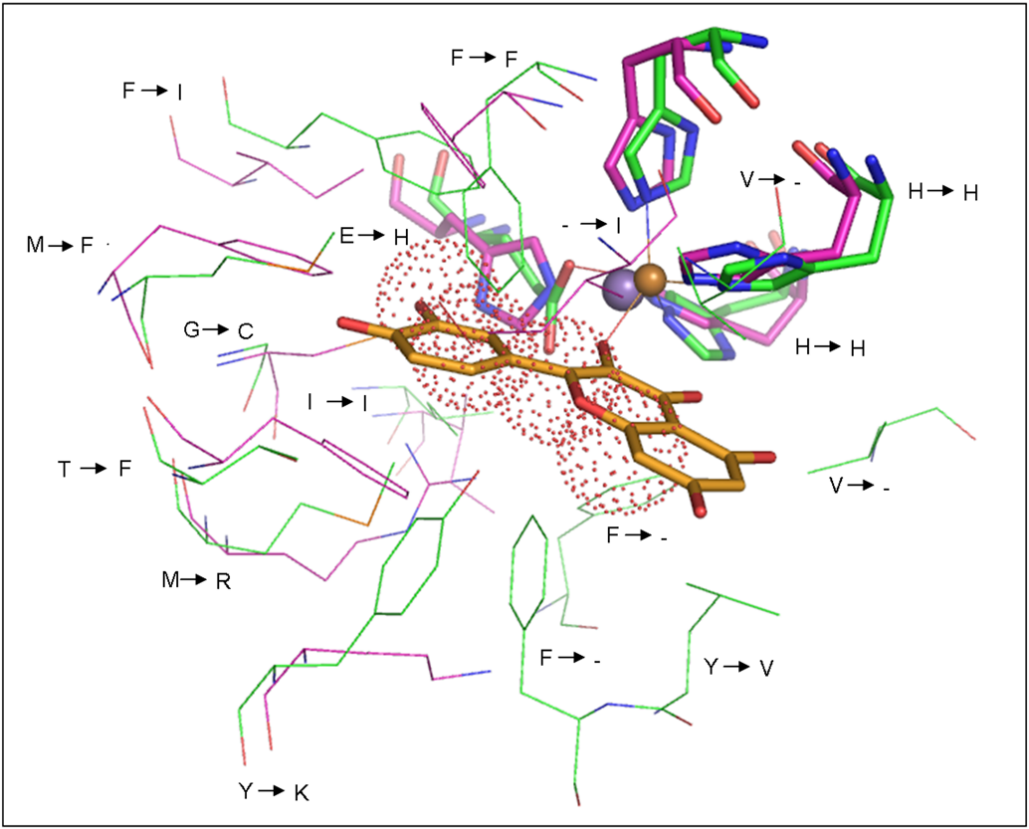
The equivalent residues at the active site region. The superposed structures of hypothetical protein (1VJ2a_, magenta) on Quercetinase (1H1Ia1, green) have been shown. The representation of metal, residues and substrates are similar to that of the previous figure.

Since proteins with similar functions have clustered together in the structure-based phylogeny, the clustering of hypothetical proteins with those of known function would provide insight into their possible functions. This has been demonstrated by considering a cluster containing two hypothetical proteins (1VJ2a_ and Bacba2) and a protein of known function, namely, quercetin dioxygenase/quercetinase [10] (1H1Ia1) (Figure 2). Although the two unannotated proteins share an overall low sequence identity to the enzyme, the residues at the active site are generally similar. The metal binding residues are either conserved or conservatively substituted hence preserving the ability of the proteins to bind metal ion. The exact details of the residues in the binding pocket and the nature of substitution of residues between quercetinase and the hypothetical proteins can be noted from Figure 8 and Figure 9 which shows the superposition of the active sites for Bacba2 and 1VJ2a_ proteins with 1H1Ia1 respectively. From figure 8 it is clear that the bulky side chains in 1H1a1 have been replaced by shorter aliphatic chains in BacBa2 indicating the possibility of accommodation of a bulkier substrate of similar nature in the hypothetical protein. In 1VJ2a_ (Figure 9), most of the substitutions are conservative except for few, such as tyrosine to lysine and methioine to arginine, which are drastic. Nevertheless, these substituted residues can interact with a similar substrate through cation-π interactions or salt bridges with polar groups on the substrate.

Although, the above examples indicate the possibility of annotation of hypothetical proteins clustering closely to the proteins of known function, this extrapolation of function based on clustering may not always hold true. An example is the clustering of hypothetical protein 1B8Ma_from an archaebacteria and 1LRHa_, an auxin binding protein known to occur in plants. The metal binding residues are not conserved. The aromatic residues in the binding pocket have been replaced by aliphatic residues, suggesting a likely aliphatic scaffold of the substrate unlike auxin (not shown). Also, the function of the archaebacterial protein, as a regulator of cell division and differentiation as in plants, is less likely. Thus, structure-based phylogeny can sometimes be used as a preliminary indicator of function of hypothetical proteins.

### Conclusions

We have demonstrated that, in general, structure-based clustering of members of cupin superfamily reflects a function-based clustering. In the cases of two cupin domains within a polypeptide chain (bicupins) we notice that the N and C-terminal cupin domains generally, cluster separately suggesting independent evolution of N and C-terminal cupin domains. The clustering of domains of unknown function indicates a conserved function among these bicupin homologues. Experimental identification of function of one such domain might help in inferring the functions of clustered domains.

Cupins have evolved functional diversity through variations in the lengths of â-strands and greater conformational freedom through loops in the barrel holding metal binding residues. Large structural variations are observed in the region interacting with another subunit or a tethered domain. This region is involved in binding to the substrate in the same domain or in the interacting one. Thus, there seems to be a complex interplay of domain tethering, quaternary states and function.

Structure-based clustering of uncharacterized proteins within a clade of proteins of known function can sometimes provide clues about their possible functions. It thus appears likely that this procedure would be a valuable tool for the functional annotation of structural genomic target proteins that are similar in structure despite poor sequence similarity.

## Materials and Methods

### Dataset sources

The current information on known structures of cupins and their classification has been obtained from the SCOP database (version 1.73) [2]. The coordinates of the protein structures were obtained from Protein Data Bank (PDB). Table 1 lists the proteins considered for the analyses along with the information on SCOP family, domain organization, quaternary states and their functions. A total of 52 proteins comprising of 76 cupin domains were considered. The lengths of the domains vary between 70 and 175 residues.

### Comparison of structures

DALI, a pairwise structural alignment algorithm [11], has been used for the alignment of protein domains with cupin fold. Simultaneous rigid-body structural superposition was performed using a robust multiple structural alignment algorithm MUSTANG [12].

### Evolutionary analysis using a structural divergence measure

Kitsch, a distance based algorithm from PHYLIP suite of programs was used for the generation of dendrograms [13]. The dendrogram has been rendered using Dendroscope software [14]. The individual domains of the proteins were considered for the phylogenetic analyses. An all against all pairwise comparison of these modules (domains) with cupin fold was performed using DALI algorithm. From the pairwise alignments, a distance matrix was computed in order to generate the dendrogram. A measure called structural distance metric (SDM), calculated as follows [15], was used to obtain the distance matrix. SDM can be defined as:
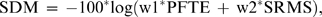
where, w1 = (1-PFTE+1-SRMS)/2 and w2 = (PFTE+SRMS)/2
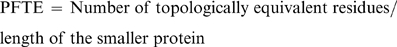


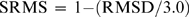
RMSD is the root mean square deviation in Å.

The calculation of SDM includes parameters that account for indels as well as root mean square deviation at the topologically equivalent positions. The SDM values therefore provide a good measure to understand the extent of differences between the structures.

## Supporting Information

Text S1 (0.05 MB DOC)

Figure S1 Reaction mechanism of quercetinase and quercetin analogues. A. Schematic representation of quercetinase activity, where quercetin is converted into 2-protocatechuoylphloroglucinol carboxylic acid. B. Variations in the substrate analogues of quercetin. Changes in the positions of the hydroxyl groups in the A and B rings are highlighted.
(0.10 MB TIF)

Figure S2 Sequence alignment of characterized quercetinases from different organisms. B. subtilis (BACSU), A. japonicus (ASPJA) and P. olsonii (PENOL) are bicupins, but Streptomyces sp. FLA (STRSP) is a monocupin. This figure shows the sequence alignment of the C-terminal domain B. subtilis with the N-terminal domains of A. japonicus and P. olsoni enzymes. The residues highlighted in grey are involved in metal ion coordination and the tyrosine residues shown in magenta interact with the oxygen in the C - ring of quercetin.
(1.60 MB TIF)

Table S1 Summary of the kinetic parameters of Quercetin and its analogues
(0.03 MB DOC)

## References

[1] Dunwell JM, Culham A, Carter CE, Sosa-Aguirre CR, Goodenough PW (2001). Evolution of functional diversity in the cupin superfamily.. Trends Biochem Sci.

[2] Murzin AG, Brenner SE, Hubbard T, Chothia C (1995). SCOP: a structural classification of proteins database for the investigation of sequences and structures.. J Mol Biol.

[3] Dunwell JM, Purvis A, Khuri S (2004). Cupins: the most functionally diverse protein superfamily?. Phytochemistry.

[4] Finn RD, Tate J, Mistry J, Coggill PC, Sammut SJ (2008). The Pfam protein families database.. Nucleic Acids Res.

[5] Balaji S, Srinivasan N (2001). Use of a database of structural alignments and phylogenetic trees in investigating the relationship between sequence and structural variability among homologous proteins.. Protein Eng.

[6] Gopal B, Madan LL, Betz SF, Kossiakoff AA (2005). The crystal structure of a quercetin 2,3-dioxygenase from Bacillus subtilis suggests modulation of enzyme activity by a change in the metal ion at the active site(s).. Biochemistry.

[7] Dunwell JM, Khuri S, Gane PJ (2000). Microbial relatives of the seed storage proteins of higher plants: conservation of structure and diversification of function during evolution of the cupin superfamily.. Microbiol Mol Biol Rev.

[8] Lichtarge O, Bourne HR, Cohen FE (1996). An evolutionary trace method defines binding surfaces common to protein families.. J Mol Biol.

[9] Mizuguchi K, Deane CM, Blundell TL, Johnson MS, Overington JP (1998). JOY: protein sequence-structure representation and analysis.. Bioinformatics.

[10] Steiner RA, Kalk KH, Dijkstra BW (2002). Anaerobic enzyme.substrate structures provide insight into the reaction mechanism of the copper-dependent quercetin 2,3-dioxygenase.. Proc Natl Acad Sci U S A.

[11] Holm L, Sander C (1993). Protein structure comparison by alignment of distance matrices.. J Mol Biol.

[12] Konagurthu AS, Whisstock JC, Stuckey PJ, Lesk AM (2006). MUSTANG: a multiple structural alignment algorithm.. Proteins.

[13] Felsenstein J (2005).

[14] Huson DH, Richter DC, Rausch C, Dezulian T, Franz M (2007). Dendroscope: An interactive viewer for large phylogenetic trees.. BMC Bioinformatics.

[15] Johnson MS, Sutcliffe MJ, Blundell TL (1990). Molecular anatomy: phyletic relationships derived from three-dimensional structures of proteins.. J Mol Evol.

[16] De Lano W (2002). The PyMOL Molecular Graphics System.

[17] Giraud MF, Leonard GA, Field RA, Berlind C, Naismith JH (2000). RmlC, the third enzyme of dTDP-L-rhamnose pathway, is a new class of epimerase.. Nat Struct Biol.

[18] Christendat D, Saridakis V, Dharamsi A, Bochkarev A, Pai EF (2000). Crystal structure of dTDP-4-keto-6-deoxy-D-hexulose 3,5-epimerase from Methanobacterium thermoautotrophicum complexed with dTDP.. J Biol Chem.

[19] Dong C, Major LL, Allen A, Blankenfeldt W, Maskell D (2003). High-resolution structures of RmlC from Streptococcus suis in complex with substrate analogs locate the active site of this class of enzyme.. Structure.

[20] Dong C, Major LL, Srikannathasan V, Errey JC, Giraud MF (2007). RmlC, a C3′ and C5′ carbohydrate epimerase, appears to operate via an intermediate with an unusual twist boat conformation.. J Mol Biol.

[21] Merkel AB, Temple GK, Burkart MD, Losey HC, Beis K (2002). Purification, crystallization and preliminary structural studies of dTDP-4-keto-6-deoxy-glucose-5-epimerase (EvaD) from Amycolatopsis orientalis, the fourth enzyme in the dTDP-L-epivancosamine biosynthetic pathway.. Acta Crystallogr D Biol Crystallogr.

[22] Swan MK, Solomons JT, Beeson CC, Hansen T, Schonheit P (2003). Structural evidence for a hydride transfer mechanism of catalysis in phosphoglucose isomerase from Pyrococcus furiosus.. J Biol Chem.

[23] McMullan D, Schwarzenbacher R, Jaroszewski L, von Delft F, Klock HE (2004). Crystal structure of a novel Thermotoga maritima enzyme (TM1112) from the cupin family at 1.83 A resolution.. Proteins.

[24] Jaroszewski L, Schwarzenbacher R, von Delft F, McMullan D, Brinen LS (2004). Crystal structure of a novel manganese-containing cupin (TM1459) from Thermotoga maritima at 1.65 A resolution.. Proteins.

[25] McLuskey K, Cameron S, Hammerschmidt F, Hunter WN (2005). Structure and reactivity of hydroxypropylphosphonic acid epoxidase in fosfomycin biosynthesis by a cation- and flavin-dependent mechanism.. Proc Natl Acad Sci U S A.

[26] Opaleye O, Rose RS, Whittaker MM, Woo EJ, Whittaker JW (2006). Structural and spectroscopic studies shed light on the mechanism of oxalate oxidase.. J Biol Chem.

[27] Woo EJ, Marshall J, Bauly J, Chen JG, Venis M (2002). Crystal structure of auxin-binding protein 1 in complex with auxin.. Embo J.

[28] Lawrence MC, Izard T, Beuchat M, Blagrove RJ, Colman PM (1994). Structure of phaseolin at 2.2 A resolution. Implications for a common vicilin/legumin structure and the genetic engineering of seed storage proteins.. J Mol Biol.

[29] Ko TP, Day J, McPherson A (2000). The refined structure of canavalin from jack bean in two crystal forms at 2.1 and 2.0 A resolution.. Acta Crystallogr D Biol Crystallogr.

[30] Adachi M, Takenaka Y, Gidamis AB, Mikami B, Utsumi S (2001). Crystal structure of soybean proglycinin A1aB1b homotrimer.. J Mol Biol.

[31] Maruyama N, Adachi M, Takahashi K, Yagasaki K, Kohno M (2001). Crystal structures of recombinant and native soybean beta-conglycinin beta homotrimers.. Eur J Biochem.

[32] Adachi M, Kanamori J, Masuda T, Yagasaki K, Kitamura K (2003). Crystal structure of soybean 11S globulin: glycinin A3B4 homohexamer.. Proc Natl Acad Sci U S A.

[33] Maruyama Y, Maruyama N, Mikami B, Utsumi S (2004). Structure of the core region of the soybean beta-conglycinin alpha' subunit.. Acta Crystallogr D Biol Crystallogr.

[34] Anand R, Dorrestein PC, Kinsland C, Begley TP, Ealick SE (2002). Structure of oxalate decarboxylase from Bacillus subtilis at 1.75 A resolution.. Biochemistry.

[35] Pang H, Bartlam M, Zeng Q, Miyatake H, Hisano T (2004). Crystal structure of human pirin: an iron-binding nuclear protein and transcription cofactor.. J Biol Chem.

[36] Cleasby A, Wonacott A, Skarzynski T, Hubbard RE, Davies GJ (1996). The x-ray crystal structure of phosphomannose isomerase from Candida albicans at 1.7 angstrom resolution.. Nat Struct Biol.

[37] Titus GP, Mueller HA, Burgner J, Rodriguez De Cordoba S, Penalva MA (2000). Crystal structure of human homogentisate dioxygenase.. Nat Struct Biol.

[38] Pochapsky TC, Pochapsky SS, Ju T, Hoefler C, Liang J (2006). A refined model for the structure of acireductone dioxygenase from Klebsiella ATCC 8724 incorporating residual dipolar couplings.. J Biomol NMR.

[39] Xu Q, Schwarzenbacher R, Krishna SS, McMullan D, Agarwalla S (2006). Crystal structure of acireductone dioxygenase (ARD) from Mus musculus at 2.06 angstrom resolution.. Proteins.

[40] Crowther RL, Georgiadis MM (2005). The crystal structure of 5-keto-4-deoxyuronate isomerase from Escherichia coli.. Proteins.

[41] Raymond S, Tocilj A, Ajamian E, Li Y, Hung MN (2005). Crystal structure of ureidoglycolate hydrolase (AllA) from Escherichia coli O157:H7.. Proteins.

[42] Zhou CZ, Meyer P, Quevillon-Cheruel S, De La Sierra-Gallay IL, Collinet B (2005). Crystal structure of the YML079w protein from Saccharomyces cerevisiae reveals a new sequence family of the jelly-roll fold.. Protein Sci.

[43] McCoy JG, Bailey LJ, Bitto E, Bingman CA, Aceti DJ (2006). Structure and mechanism of mouse cysteine dioxygenase.. Proc Natl Acad Sci U S A.

[44] Zhang Y, Colabroy KL, Begley TP, Ealick SE (2005). Structural studies on 3-hydroxyanthranilate-3,4-dioxygenase: the catalytic mechanism of a complex oxidation involved in NAD biosynthesis.. Biochemistry.

