# Supplementary material for: Structure-Based Phylogeny as a Diagnostic for Functional Characterization of Proteins with a Cupin Fold
Source: PLoS One. 2009 May 29;4(5):e5736. doi: 10.1371/journal.pone.0005736 (PMC2684688; doi:10.1371/journal.pone.0005736)
Supplement: Text S1 — (0.05 MB DOC) [file pone.0005736.s001.doc]

**Supplementary Information**

Corresponding to the paper entitled

**“Structure-Based Phylogeny as a Diagnostic for Functional Characterization of Proteins with a Cupin Fold”**

by

G. Agarwal, M. Rajavel, B. Gopal & N. Srinivasan

**Inference on substrate specificity for proteins that are closely clustered in the structure-based phylogeny: A Case Study on Quercetinases**

Quercetinases catalyze the cleavage of the O- heteroaromatic ring of the flavonol quercetin (5, 7, 3’, 4’- tetrahydroxy flavonol) to 2-protocatechuolyphlororglucinol carboxylic acid and carbon monoxide (Figure S1A). In this reaction, the triplet ground state of O_2_ reacts with the singlet ground state of quercetin to give the product with the release of CO. As this reaction is a spin forbidden process, activation of Fe^2+^ prior to the reaction is essential. In the presence of free radical to initiate the reaction, the hydrogen atom from position 3 of the flavonol ring is abstracted. An alternative is the formation of a flavonoxy radical by the transfer of an electron from the flavonol to the Fe^2+^. The major difference between the natural reaction and the enzyme-catalyzed reaction therefore lies in the way the flavonoxy radical is subjected to attack. In an effort to determine if this reaction mechanism could be influenced by the positioning of the substrate in the active-site cavity, analogues of quercetin (Figure S1B) were examined. All the kinetic parameters including Kcat/Km values corresponding to dioxygenase activity using quercetin and its analogues are given in Table S1. That substrate specificity is hard-wired is clearly seen by the enzyme kinetics parameters. The order of the substrate preferences (Figure S1B and Table S1) show that quercetin is the most suited and fisetin is the least favored substrate amongst the analogues that were examined in this study. The flavonol morin, which differs from quercetin in the B ring with a hydroxyl group located at the 2’position, appears to be the next most suited substrate for the *B. subtilis* enzyme. The inability of the enzyme to oxidize the quercetin analogue 7-Hydroxy flavone is consistent with the model proposed for the catalytic mechanism of quercetinase. Apart from the positional specificity of the hydroxyl group at position 2 of the hetero-ring for quercetinase activity, the hydroxyl group at position 5 of the A ring is seen to be the next important substitution. Changes in the A ring of the flavonol are tolerated to a much greater extent than changes in the hetero-ring. One likely reason for this finding could be that variations in the A ring can be accommodated by the deformation of the substrate that occurs upon binding [1]. Another interesting observation in this study is the involvement of oxygen atom in the C-ring (C1) of quercetin and its analogues in positioning the substrates in the active site. A proximal tyrosine residue in quercetinase interacts through a hydrogen bond with this oxygen atom. A multiple sequence alignment of known quercetinases shown in Figure S2, is consistent with the role of tyrosine in positioning the substrate in the active site. The residues highlighted in grey are involved in metal ion coordination and the tyrosine residues shown in magenta.

An analysis of the *B. subtilis* quercetinase structure and the molecular model of the enzyme-quercetin complex suggest that the structural features that govern quercetinase-substrate interactions are conserved between the *A. japonicus* quercetinase and the *B. subtilis* homologue. For example, in the case of *A. japonicus* enzyme, Met 51, Thr 53, Phe 75, Phe 114 and Met 123 interact with the ‘B’ ring of quercetin as well as Gly125 through van der Waals interactions[1]. These residues are mostly conserved in the *B. subtilis* homologue. These sequence and structural similarities allowed us to use the model of the *B. subtilis* enzyme-quercetin complex to understand the interaction of this quercetinase with quercetin and analogues. The crystal structure of the *A. japonicus* quercetinase-substrate complex revealed a deformation of quercetin upon binding to the active site cavity. This deformation in the substrate was suggested as a mechanism that could lower the activation energy for the oxidation reaction [1]. The loop connecting the two domains was also shown to play a role in stabilizing the substrate in the active site cavity. Another interesting observation on the reaction mechanism comes from Molecular Dynamics simulations on this protein [2,3]. These theoretical studies suggest that quercetinase has two distinct entry sites dedicated to the flavonoid substrate and the dioxygen moiety, leading to the conclusion that this enzyme is structurally optimized to bind the two chemical entities needed in this biochemical reaction[2]. It is thus likely that in the expansion of the functional repertoire within the bicupin scaffold, improvisations such as an addition of hydrophobic channels that could allow the passage of small hydrophobic ligands such as O_2_ could also lead to the optimization of a particular enzymatic function.

**Materials and Methods**

#### Quercetinase assays with the flavonol quercetin and its analogues:

10 mM stocks of quercetin and other substrate analogues were prepared in Dimethyl Sulphoxide (DMSO). Enzyme assays to determine the Km and Vmax values of the quercetinase with various substrates were carried out in 25 mM Tris-Cl buffer pH 7.5. The reaction conditions had 1µg (0.017µM) of the enzyme (in a total volume of 1500µl) with substrate concentrations ranging from 1µM to 40µM. The activity assays were monitored by a decrease in absorption values of the substrate at the max of substrate absorption. The ε values used to calculate the substrate concentrations were: Quercetin ε367 = 17200 M^-1^cm^-1^, Kaempferol ε363 = 11900 M^-1^cm^-1^, Myricetin ε375 = 16200 M^-1^cm^-1^, Morin ε388 = 13400 M^-1^cm^-1^, Fisetin ε370 = 20000 M^-1^cm-1 and 7-Hydroxy flavone ε310 =15500 M^-1^cm^-1^. Absorption readings were monitored at 30 sec. intervals for a total of 300 sec. on a Jasco UV-Visible Spectrophotometer (Jasco, Inc). A Michaelis-Menten plot to obtain the Km and Vmax of the quercetinase with quercetin and its analogues was calculated using the SIGMA PLOT tool (Systat Software Inc.).

**References to supplementary material**

1. Tranchimand S, Ertel G, Gaydou V, Gaudin C, Tron T, et al. (2008) Biochemical and molecular characterization of a quercetinase from Penicillium olsonii. Biochimie 90: 781-789.

2. Bowater L, Fairhurst SA, Just VJ, Bornemann S (2004) Bacillus subtilis YxaG is a novel Fe-containing quercetin 2,3-dioxygenase. FEBS Lett 557: 45-48.

3. Merkens H, Sielker S, Rose K, Fetzner S (2007) A new monocupin quercetinase of Streptomyces sp. FLA: identification and heterologous expression of the queD gene and activity of the recombinant enzyme towards different flavonols. Arch Microbiol 187: 475-487.

| **Table S1: Summary of the kinetic parameters of Quercetin and its analogues** | | | | | |
| --- | --- | --- | --- | --- | --- |
| **Substrate** | **- OH position** | **Vmax (U/mg)** | **Km(μM)** | **Kcat(s^-1^)** | **Kcat/Km**  **(μM^-1^ s^-1^)** |
| Quercetin | 3’,4’,6,8 | 1.84 | 1.48±0.08 | 1.23 | 0.83±0.04  (8.8[1])  (0.10[3])  (0.21[2]) |
| Morin | 2’,4’,6,8 | 0.42 | 0.76±0.14 | 0.41 | 0.54±0.09 |
| Kaempferol | 4’,6,8 | 5.97±1.05 | 6.24±4.21 | 5.85 | 0.094±0.63  (36[1]) |
| Myricetin | 3’,4’,5’,6,8 | 1.99±0.1 | 14.03±1.52 | 1.95±0.09 | 0.14±0.01 |
| Fisetin | 3’,4’,8 | 0.19±0.01 | 2.74±0.74 | 0.19±0.01 | 0.07±0.02  (0.67[1]) |
| 7-Hydroxy Flavone | 8 | inactive | | | |
