# Supplementary figures and images for: Structure-Based Phylogeny as a Diagnostic for Functional Characterization of Proteins with a Cupin Fold
Source: PLoS One. 2009 May 29;4(5):e5736. doi: 10.1371/journal.pone.0005736 (PMC2684688; doi:10.1371/journal.pone.0005736)

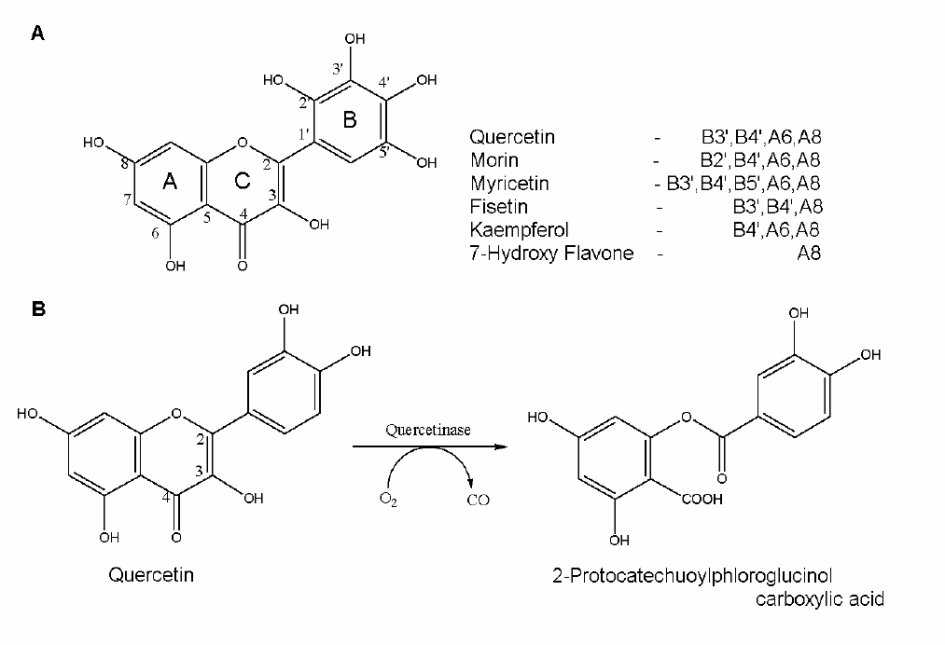

Supplement: Figure S1 — Reaction mechanism of quercetinase and quercetin analogues. A. Schematic representation of quercetinase activity, where quercetin is converted into 2-protocatechuoylphloroglucinol carboxylic acid. B. Variations in the substrate analogues of quercetin. Changes in the positions of the hydroxyl groups in the A and B rings are highlighted. (0.10 MB TIF) [file pone.0005736.s002.tif]

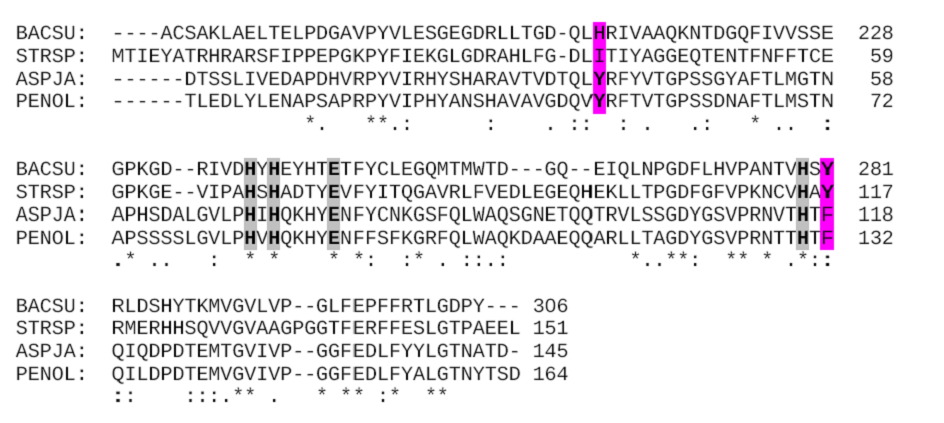

Supplement: Figure S2 — Sequence alignment of characterized quercetinases from different organisms. B. subtilis (BACSU), A. japonicus (ASPJA) and P. olsonii (PENOL) are bicupins, but Streptomyces sp. FLA (STRSP) is a monocupin. This figure shows the sequence alignment of the C-terminal domain B. subtilis with the N-terminal domains of A. japonicus and P. olsoni enzymes. The residues highlighted in grey are involved in metal ion coordination and the tyrosine residues shown in magenta interact with the oxygen in the C - ring of quercetin. (1.60 MB TIF) [file pone.0005736.s003.tif]
