# Supplementary material for: Structure-Based Phylogeny as a Diagnostic for Functional Characterization of Proteins with a Cupin Fold
Source: PLoS One. 2009 May 29;4(5):e5736. doi: 10.1371/journal.pone.0005736 (PMC2684688; doi:10.1371/journal.pone.0005736)
Supplement: Table S1 — Summary of the kinetic parameters of Quercetin and its analogues (0.03 MB DOC) [file pone.0005736.s004.doc]

| **Table S1: Summary of the kinetic parameters of Quercetin and its analogues** | | | | | |
| --- | --- | --- | --- | --- | --- |
| **Substrate** | **- OH position** | **Vmax (U/mg)** | **Km(μM)** | **Kcat(s^-1^)** | **Kcat/Km**  **(μM^-1^ s^-1^)** |
| Quercetin | 3’,4’,6,8 | 1.84 | 1.48±0.08 | 1.23 | 0.83±0.04  (8.8[1])  (0.10[3])  (0.21[2]) |
| Morin | 2’,4’,6,8 | 0.42 | 0.76±0.14 | 0.41 | 0.54±0.09 |
| Kaempferol | 4’,6,8 | 5.97±1.05 | 6.24±4.21 | 5.85 | 0.094±0.63  (36[1]) |
| Myricetin | 3’,4’,5’,6,8 | 1.99±0.1 | 14.03±1.52 | 1.95±0.09 | 0.14±0.01 |
| Fisetin | 3’,4’,8 | 0.19±0.01 | 2.74±0.74 | 0.19±0.01 | 0.07±0.02  (0.67[1]) |
| 7-Hydroxy Flavone | 8 | inactive | | | |
